# Non-invasive health prediction from visually observable features

**DOI:** 10.12688/f1000research.72894.2

**Published:** 2022-03-02

**Authors:** Fan Yi Khong, Tee Connie, Michael Kah Ong Goh, Li Pei Wong, Pin Shen Teh, Ai Ling Choo

**Affiliations:** 1 Faculty of Information Science and Technology, Multimedia University, Melaka, Melaka, 75450, Malaysia; 2 School of Computer Sciences, Universiti Sains Malaysia, Penang, Penang, 11800, Malaysia; 3 Department of Operations, Technology, Events and Hospitality Management, Faculty of Business and Law, Manchester Metropolitan University, Manchester, Manchester, M15 6BH, UK; 4 iRadar Sdn. Bhd., Melaka, Melaka, 75450, Malaysia

**Keywords:** Machine learning, Health prediction, Remote screening and diagnosis

## Abstract

**Background: **The unprecedented development of Artificial Intelligence has revolutionised the healthcare industry. In the next generation of healthcare systems, self-diagnosis will be pivotal to personalised healthcare services. During the COVID-19 pandemic, new screening and diagnostic approaches like mobile health are well-positioned to reduce disease spread and overcome geographical barriers. This paper presents a non-invasive screening approach to predict the health of a person from visually observable features using machine learning techniques. Images like face and skin surface of the patients are acquired using camera or mobile devices and analysed to derive clinical reasoning and prediction of the person’s health.

**Methods: **In specific, a two-level classification approach is presented. The proposed hierarchical model chooses a class by training a binary classifier at the node of the hierarchy. Prediction is then made using a set of class-specific reduced feature set.

**Results:** Testing accuracies of 86.87% and 76.84% are reported for the first and second-level classification. Empirical results demonstrate that the proposed approach yields favourable prediction results while greatly reduces the computational time.

**Conclusions: **The study suggests that it is possible to predict the health condition of a person based on his/her face appearance using cost-effective machine learning approaches.

## Introduction

As technology advances, machine learning techniques have been growing in popularity over the past years. Machine learning techniques have proven to be effective in solving many modern problems in different domains. There is an increased research interest in applying machine learning methods for clinical informatics and healthcare systems. ^1- 4^ Meanwhile, facial recognition technology has been vastly utilized in various fields. For instance, it has been applied to unlock phones, find wanted fugitives and diagnose diseases. There have been many kinds of research done on disease diagnosis using facial images. ^5- 8^ Systems that only use facial features to diagnose illnesses are beneficial for remote medical diagnosis.

In this research, a machine learning approach was developed to detect the health condition of a person based on facial features. The purpose of the health prediction system was to identify images as ‘healthy’, or ‘ill’ with either ‘fever’, ‘sore throat’, or ‘runny nose’ symptoms. Facial images containing healthy and ill faces (fever, sore throat and runny nose) were collected. Then, discriminative facial features were extracted from the images using different feature extraction techniques. These features were used to train several machine learning classifiers for health prediction.

### Literature review

In this section, various types of approaches to health prediction using facial features are studied and reviewed to learn about their respective advantages and disadvantages. These approaches are separated into two categories: conventional approaches and deep learning approaches.

*Conventional approaches*

In 2013, Zhao *et al.* ^1^ introduced an approach to classify Down Syndrome through image-based facial dysmorphology. Facial features were extracted using Contourlet transform-based and local binary pattern- based (LBP) local texture features, as well as geometric features using landmarks of facial anatomy. The support vector machine (SVM) classifier, this technique has produced an accuracy of 97.92%.

A survey done by ^2^ about genetic disorders diagnosis based on facial images, Saraydemir *et al.* ^3^ presented an approach to identify subjects with Down Syndrome from healthy subjects using facial image. Gabor wavelet transform (GWT) was implemented for feature extraction purposes. Then, linear discriminant analysis (LDA) and principal component analysis (PCA) were carried out for the reduction of dimension. 96% and 97.34% accuracy were produced.

A research conducted by ^5^ developed an approach for identifying Down Syndrome based on analysis of facial landmarks on 2D images. An independent component analysis-based hierarchical constrained local model (HCLM) was introduced to identify the landmarks of a face. The method was also tested on a mixed-syndromes dataset, and the highest accuracy achieved was 97%.

Another study related to health prediction systems using facial features that uses traditional machine learning methods, is an acromegaly identification using facial images proposed by. ^6^ A few conventional methods such as SVM, generalized linear models (LM), k-NN, RF of randomized trees (RT) as well as other deep learning methods were used to train the model. The best performance was attained by the SVM method with a 95% PPV and 88% NPV, and with an accuracy of 91%. With frontalized faces, k-NN worked best with 89% PPV and 93% NPV, also with an accuracy of 91%.

*Deep learning approaches*

In 2018, Sajid *et al.* ^7^ developed a palsy grading system based on unsymmetrical facial features using deep learning. A convolutional neural network (CNN) was proposed to extract features that exhibited palsy symptoms from a large number of facial images. The results of the model on the improved dataset showed a recognition rate of 92.6%.

A facial analysis framework introduced by ^8^ called DeepGestalt, to identify rare genetic syndromes using deep learning. The training process of the DeepGestalt model consisted of two steps. Firstly, an overall representation of the face was learned by the model. The binary classification problem of identifying Angelman Syndrome (AS) and Cornelia De Lange Syndrome (CdLS) patients achieved an accuracy of 92% and 96.88%, respectively.

In year 2020, ^9^ proposed to detect cancer using the facial features of patients. They used the network architecture of a residual neural network (ResNet) which comprised 27 convolution layers and two fully connected (FC) layers. Transfer learning was also applied for convolution layers 1-5 by directly obtaining the weights from a pre-trained face encoding model developed by. ^10^ To describe the distinguishing traits of non-cancer and cancer datasets, they used gradient-weighted class activation mapping (grad-CAM) for the model that they trained. The accuracy rate produced by this approach was 82%.

Apart from that, ^11^ developed a technique to detect Down Syndrome automatically based on facial images with deep convolutional neural network (DCNN). Firstly, they trained a DCNN on a large dataset to acquire an overall face encoding network. The network architecture consists of ten convolutional layers activated by ReLU along with three FC layers. This method achieved an accuracy of 95.87%.

Also in 2020, ^12^ developed a study to diagnose and classify the severity of acromegaly at different severity levels using facial images with deep learning. CNN was used in this method. For facial recognition, the pre-trained Inception ResNet V1 was utilized to extract features. The total prediction accuracy achieved by this method was 90.7%.

Besides, Forte *et al.* ^13^ presented a deep learning approach to assess a patient’s health by using facial and bodily cues. To increase the dataset size, a synthetic dataset containing acutely ill images were generated using a neural transfer CNN network. After that, four CNN models were trained on different parts of the faces and the features were concatenated into a final feature and fed to a staked CNN. The proposed model was tested using a dataset that was made up of images of volunteers injected with lipopolysaccharide.

On the other hand, Onyema *et al.* ^14^ performed facial recognition for patients monitoring using ResNet. Facial emotions is believed to be closely related to the patient’s state of mind. The seven universal emotions including happy, sad, fear, anger, surprise and neural were investigated. Data augmentation was applied to increase the diversity of the data. An accuracy of 70% was achieved using the proposed approach.

Recently, Connie *et al.* ^15^ proposed an explainable AI approach for providing explanations for the predictions made by an AI model for health application. A transfer learning approach with VGGFace model was applied to process the facial images. After that, an outcome whether the face belongs to a sick person was derived. Explainable AI (XAI) was used to provide explanation why the outcome, e.g. sick or healthy face, was produced. Different XAI techniques including Integrated Gradient, Explainable region-based AI (XRAI) and Local Interpretable Model-Agnostic Explanations (LIME) were investigated in the paper. The proposed approach had helped to increase the accountability of the healthcare system. A summary of works related to this study (hand-crafted features based methods), together with the pros and cons of each method, is presented in Table 1.

**Table 1.** A summary of works related to this study.

Author	Method	Database	Classes	Recognition Rate	Pros	Cons
Zhao, Q., Rosenbaum, K., Sze, R., Zand, D., Summar, M., & Linguraru, M. G. ^1^	▪ Geometric + SVM ▪ Texture + SVM ▪ Combined + SVM	Self-collected dataset	Down syndrome + Normal	97.92%	1. Contourlets preserve important wavelet features and provide a high level of anisotropy and directionality 2. LBP features are robust against illumination changes and takes less computational time	Facial anatomical landmarks and texture features need to be defined manually, requires more time and effort
Saraydemir, Ş., Taşpınar, N., Eroğul, O., Kayserili, H., & Dinçkan, N. ^3^	▪ GWT + PCA & LDA + SVM ▪ GWT + PCA & LDA + k-NN	▪ University Medicine Faculty Department of Medical Genetics ▪ Down Syndrome Association of Turkey and Istanbul	Down syndrome + Healthy	97.34%	1. Dataset is small to produce robust results 2. Resistant to biases due to pose, illumination, and expression variances	Manual normalization requires more effort and time than automated approaches
Ferry, Q., Steinberg, J., Webber, C., FitzPatrick, D. R., Ponting, C. P., Zisserman, A., & Nellåker, C. ^4^	PCA + AAM + k-NN	▪ Publicly available resources ▪ Scientifically published pictures of patients	Eight genetic disorders + Healthy	99.5%	1. Robust to artificial variations such as lighting, pose, and image quality 2. Provides consistent computational descriptions of facial gestalt	1. AAMs involve complex texture mapping and image warping operations which are susceptible to errors 2. AAMs have low performance on unseen faces
Zhao, Q., Okada, K., Rosenbaum, K., Kehoe, L., Zand, D. J., Sze, R., Summar, M., & Linguraru, M. G. ^5^	Features: ▪ Geometric ▪ LBP ▪ Geometric + LBP ▪ GWT ▪ Geometric + GWT Classifiers: ▪ SVM-RBF ▪ Linear SVM ▪ k-NN ▪ RF ▪ LDA	Self-collected dataset	Down syndrome + Healthy	96.7%	1. CLMs are more generative and discriminative on unseen appearance 2. CLMs are more constant to global illumination variation and occlusion	1. ICA requires large datasets to train to produce good results 2. Optimization can converge to local minima or false locations
			Mixed syndromes + Healthy	97%		
Kong, X., Gong, S., Su, L., Howard, N., & Kong, Y. ^6^	▪ k-NN ▪ SVM ▪ RF	▪ SCUT-FBP dataset ▪ Neurosurgery inpatient departments of hospitals in China ▪ Self-collected dataset	Acromegaly + Normal	95%	SVM performs well on extracted facial features	1. A possibility of bias caused by the selection of samples may occur 2. It is not known whether the outcome is generalizable to different populations

## Methods

### Proposed solution

**Figure 1.**
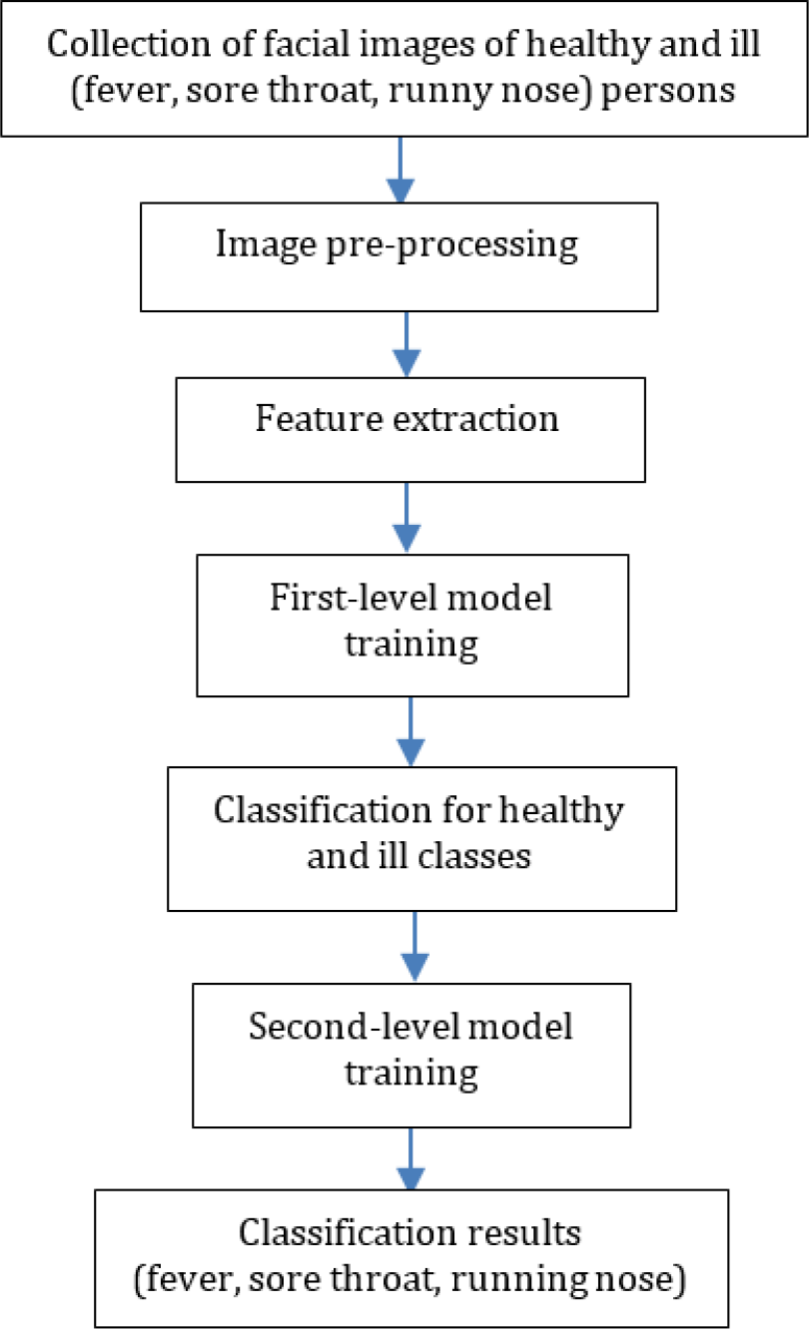
Proposed framework.

A two-level classification approach is presented in this paper for health prediction based on facial features. Figure 1 shows the processes of how a prediction model was developed. First, facial images of healthy and ill (fever, sore throat and runny nose) persons were collected. Then, these images were pre-processed to clean, standardize and normalize the data. There are two levels of classification. The first-level classification is responsible for classifying samples into ‘healthy’ and ‘ill’ classes, while the second-level classification is in charge of classifying the ‘ill’ samples’ into ‘fever’, ‘sore throat’, and ‘runny nose’ classes. Therefore, there are two levels of model training in the proposed solution. In this research, conventional machine learning methods were adopted.

The feature extraction methods used were local binary pattern (LBP), Principal Component Analysis (PCA), Linear Discriminant Analysis (LDA) and Gabor filter. LBP is a straightforward texture analysis method that constructs binary numbers by thresholding the neighbours of every pixel in an image. For every pixel, its eight neighbours are examined to see whether their intensity is higher than the particular pixel. The threshold results from the eight neighbours are used to construct an eight-digit binary number. If the intensity of the neighbour is less than or equal to the pixel, then the first digit of the binary number would be 0, otherwise, it would be 1. Then, the texture of the image is represented by a histogram of these numbers.

On the other hand, PCA is a dimensionality reduction method that works by finding out patterns and correlations that best represent the data in a least-square sense. Higher-dimensional data are projected to a lower-dimensional space. It is an unsupervised technique that does not consider labels. It seeks directions that maximize variance and are efficient for representation.

LDA is also a dimensionality reduction tool. Higher-dimensional data are projected to a lower-dimensional space. It works by finding the projection that best separates the data of two or more classes in a least-square sense. It is a supervised technique that seeks directions that maximizes the distance between classes and are efficient for discrimination.

On the contrary, Gabor filter is a technique used for texture analysis, edge detection, feature extraction and more. These filters have been claimed that they stimulate the visual system of some mammals. They can filter any particular frequencies in an image in the region of analysis. For example, they recognize some specific frequencies and ignore the rest. To analyse the texture from an image, a collection of Gabor filters containing different orientations are applied.

In this study, the pre-processed images are converted to grayscale before the features are extracted from the images. After that, the features from the images are extracted in two different ways. For LBP and Gabor filter features, the feature extraction procedure follows the order of: loading original and augmented images, extracting features from the whole dataset, separating the original and augmented images’ extracted features from the dataset, splitting the extracted features of the original dataset into training and testing sets, adding the extracted features of the augmented dataset into the training set, and shuffling, and scaling the training and testing sets (as required depending on the model’s performance).

On the other hand, for PCA and LDA features, the feature extraction procedure follows the order of: loading original and augmented images, splitting the original dataset into training and testing sets, adding the augmented dataset into the training set, and shuffling, scaling the training and testing sets (as required depending on the model’s performance), and extracting features from the training and testing sets.

The classifiers used were SVM, NN, KNN, and RF. A total of 16 combinations among the feature extraction techniques and classifiers mentioned were experimented with to find the best-performing model.

#### Datasets

In this study, a total of 733 facial images of healthy and ill persons were collected. Among 733 images, 233 are images of ill persons who had either fever, sore throat, or runny nose and 500 were images with healthy or normal persons. 420 out of the 500 healthy images contained normal faces of people from ages 1 to 50, while the remaining 80 images were healthy throat images. Images of healthy throat and ill persons were manually collected from various online sources, while images of healthy faces were obtained from the UTKFace database. ^16^ The number of images for each class and subclass is listed in Table 2.

**Table 2.** Number of images for each class and subclass.

Class	Subclass	Number of images
**Healthy**	-	500
**Il**	Fever	78
	Sore throat	80
	Running nose	75

### Results and discussion

In this section, the experimental results for the different models that consist of the combinations of four feature extraction methods and four classifiers are presented, analysed and discussed. The testing accuracies of the first and second-level classification of each model were recorded for 10 runs. ^17^

#### SVM variants

The first experiment validates the performance of the SVM variant. Table 3 demonstrates the results of the SVM variant for the first and second classification tests. Among LBP, PCA, LDA and Gabor filter features, PCA features performed the best with SVM in the first-level classification. It achieves a promising result of 85.85% average testing accuracy with minimal overfitting. On the other hand, the LBP features performed the best with SVM in the second-level classification, with an average testing accuracy of 73.32%. The SVM variants generally produced results with the least overfitting among all the classifiers. ^18^

**Table 3.** Experimental results of SVM variants.

Methods	1 ^st^ Level classification testing	2 ^nd^ Level classification testing
LBP + SVM	80.88	73.32
PCA + SVM	85.85	64.05
LDS + SVM	85.37	63.01
GABOR FILTER + SVM	81.29	63.45

#### NN variants

The experimental results for the NN variants are depicted in Table 6. Among all the feature extraction techniques, PCA features worked best with NN in the first-level classification. It achieved an average testing accuracy as high as 91.84%. On the other hand, the LBP features performed best with NN in the second-level classification with an average testing accuracy of 76.84%. In the second-level classification, the LBP model was also the only model that stood out among the other NN variants. ^19^

#### KNN variants

The performance of the KNN variants for the first and second level classifications is given in Table 5. Among all the feature extraction techniques, again, PCA features worked best with KNN in the first-level classification, with an average testing accuracy as high as 90.34%. The same model also performed best in the second-level classification among all the KNN variants, as it obtained an average testing accuracy of 70.03%.

***RF variants***

The experimental results for the RF variants are displayed in Table 6. Among all the features extraction techniques, once again, at 88.57% average testing accuracy, PCA features performed the best with RF in the first-level classification. This model also scored best in the second-level classification among all the RF variants as it obtained an average testing accuracy of 74.15%.

#### First-level classification results

According to the experimental results of all the models shown in Tables 3 to 6, two models achieved over 90% average testing accuracies in the first-level classification. These models are the PCA+NN and PCA+KNN model.

**Table 4.** Experimental results of NN variants. The best results are highlighted in bold.

Methods	1 ^st^ Level classification testing	2 ^nd^ Level classification testing
LBP + NN	86.87	**76.84**
PCA + NN	**91.84**	66.96
LDA + NN	86.87	63.19
GABOR FILTER + NN	86.05	66.93

**Table 5.** Experimental results of KNN variants. The best results are highlighted in bold.

Methods	1 ^st^ Level classification testing	2 ^nd^ Level classification testing
LBP + NN	83.54	65.33
PCA + KNN	**90.34**	**70.03**
LDA + KNN	86.53	63.78
GABOR FILTER + KNN	72.26	63.02

**Table 6.** Experimental results of RF variants. The best results are highlighted in bold.

Methods	1 ^st^ Level classification testing	2 ^nd^ Level classification testing
LBP + RF	83.61	67.95
PCA + RF	**88.57**	**74.15**
LDA + RF	85.31	64.15
GABOR FILTER + RF	87.89	62.41

*PCA+NN*

The model that achieved the highest accuracy in the first-level classification was PCA+NN. It obtained a 91.84% average testing accuracy. The high accuracy could be due to the fact that PCA effectively reduces the dimensions of data and it is able to capture the important correlations and patterns that best characterize the data. The misclassified samples were plotted during one of the runs of the finalized PCA+NN model. Out of the 147 samples, there were 15 misclassified samples.

*PCA+KNN*

**Figure 2.**
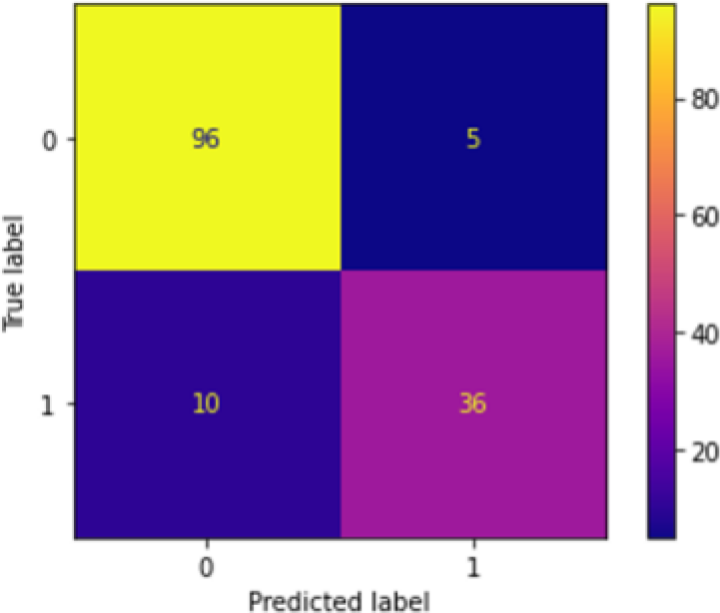
Confusion matrix of PCA+KNN at first-level classification.

The PCA+KNN model obtained the second-highest accuracies in the first-level classification after PCA+NN. Its performance was as good as that of PCA+KNN as it achieved a 90.34% average testing accuracy. Figure 2 shows the confusion matrix after running the first-level classification of PCA+KNN model. There is not much difference between the performance of PCA+NN and PCA+KNN as both of them were able to perform equally well.

*Overall first-level performance*

Apart from PCA+NN and PCA+KNN, the overall results of the first-level classification were rather good as most of the models achieved an average testing accuracy of 80% and above. Even though the other models overfit more than PCA+NN and PCA+KNN, their results were still considered rather satisfactory. The symptoms shown on the faces of ill people or sore throats are important features to help the model classify healthy and ill samples. ^20^

#### Second-level classification results

Based on the results given in Tables 3 to 6, a total of four models achieved average testing accuracies between 70% and 77% in the second-level classification. These models were the LBP+NN, PCA+RF, LBP+SVM, and PCA+KNN model.

*LBP+NN*

The model that achieved the highest accuracy in the second-level classification was LBP+NN. It obtained an average testing accuracy of 76.84%. Its performance was considered rather satisfactory, as most of the other models only obtained testing accuracies between 60% and 68% on average. The reason that LBP+NN could perform well could be that the LBP features were invariant to illumination and were highly discriminative.

*PCA+RF*

**Figure 3.**
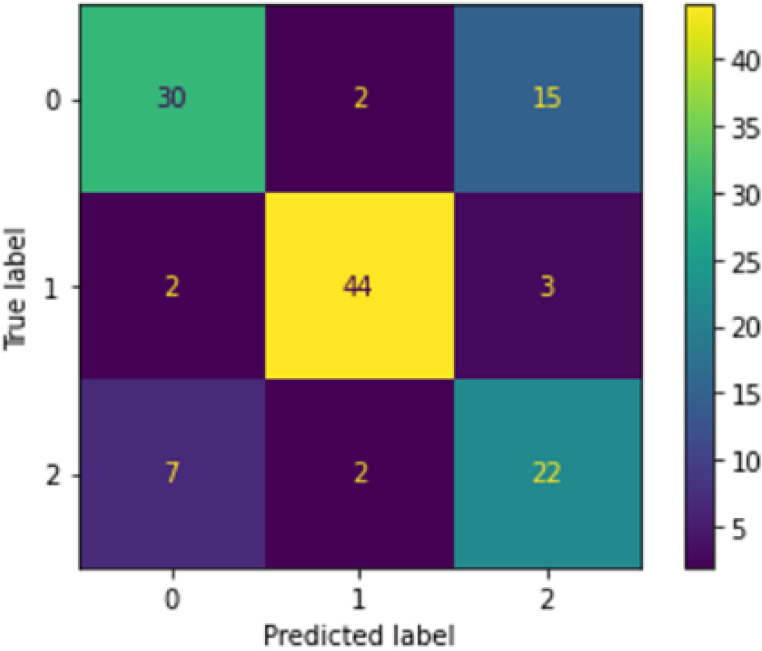
Confusion matrix of PCA+RF at second-level classification.

The PCA+RF model performed nearly as well compared to LBP+NN with an average testing accuracy of 74.15% in the second-level classification. It performed well due to the previously mentioned benefits of the combination of PCA and RF being a classifier with outstanding predictive capabilities. Figure 3 shows the confusion matrix produced after running the second-level classification of PCA+RF model.

The confusion matrix was generated during one of the runs of the finalized PCA+RF model. The 0 label represents the ‘fever’ class, 1 represents the ‘sore throat’ class and 2 represents the ‘running nose’ class. It can be seen that the top two misclassified classes were the ‘fever’ (0) and ‘runny nose’ (2) classes, with 15 fever samples misclassified as runny nose and seven runny nose samples misclassified as fever. The reason for this occurrence is the same as for the LBP+NN model’s case. The total number of samples misclassified by PCA+RF for this run was 31 samples, with only one additional misclassified sample compared to the LBP+NN model. Hence, PCA+RF was able to produce results as good as LBP+NN in the second-level classification.

*LBP+SVM and PCA+KNN*

Other than the LBP+NN and PCA+RF, the two remaining models that achieved over 70% average testing accuracies were LBP+SVM and PCA+KNN. The LBP+SVM model obtained a 73.32% average testing accuracy in the second-level classification. The reason behind its performance is the robustness of LBP as well as the fact that SVM is effective in situations where the number of dimensions is larger than the number of samples. In the model’s second-level classification, the number of testing samples was always lesser than the number of dimensions.

#### Best model for the health prediction system

Among all models, the LBP+NN variant had the best overall performance in the first and second-level classifications. It achieved the highest average testing accuracy of 76.84% in the second-level classification. It also performed considerably well in the first-level classification with lesser overfitting than the other models with similar performances, as it showed 94.38% and 86.87% average training and testing accuracies, respectively.

#### Comparison with other methods

A comparison of the proposed methods with state-of-the-art approaches is presented in Table 7. It can be observed that the deep learning approaches including CNN ^1^ and VGGFace ^2^ outperform the proposed methods that rely on hand-crafted features. Nevertheless, the proposed approach has a great advantage as compared to the deep learning approaches in terms of computational speed. For example, it only took 0.0015 seconds to train the PCA+RF classifier, while it takes more than five minutes to perform training using the deep learning models. Therefore given a scenario where speed is a critical requirement and there is not many training samples available, the proposed method appears to be a more favourable choice.

**Table 7.** A comparison with state-of-the-art methods.

Methods	1 ^st^ Level classification testing
LBP + NN (First level classification)	86.87
PCA + RF (First level classification)	88.57
CNN ^7^	92.71
VGGFace ^21^	96.25

### Conclusions

This paper presents a health prediction system using facial features evaluated using different machine learning models. Datasets containing facial images of healthy and ill (fever, sore throat and runny nose) persons were collected. The facial features of the images were extracted using LBP, PCA, LDA and Gabor filter feature extraction techniques. The features were trained using SVM, NN, KNN and RF classifiers. Among the 16 models, the LBP+NN model yielded the best overall performance for both the first and second-level classifications. It obtained average testing accuracies of 86.87% and 76.84% for the first and second-level classification, respectively.

### Data availability

#### Underlying data

UTKFace Large Scale Face Dataset: https://susanqq.github.io/UTKFace/.

As it is impossible to obtain the consent for the face images retrieved from the UTKFace dataset, the images cannot be shared in this article.

### Software availability

Source code available from: https://doi.org/10.5281/zenodo.5266406. ^22^

Data are available under the terms of the Creative Commons Zero “No rights reserved” data waiver (CC0 1.0 Public domain dedication).
