# Supplementary material for: Non-invasive health prediction from visually observable features
Source: F1000Res. 2022 Mar 2;10:918. Originally published 2021 Sep 13. [Version 2] doi: 10.12688/f1000research.72894.2 (PMC9039370; doi:10.12688/f1000research.72894.2)
Supplement: Peer review history

# Peer review: Reviewer response for version 2

**DOI:** 10.5256/f1000research.121466.r134514

**Published:** 2022-04-25

**Authors:** Yong Wee Sek

**Affiliations:** 1 Fakulti Teknologi Maklumat dan Komunikasi, Universiti Teknikal Malaysia Melaka, Melaka, Malaysia

In this paper, the authors introduce a machine learning approach to detect the health condition of a person based on facial features. There is a great deal of good work in this paper. However, it does require a significant overall revision to make the best of this and present the work in a clear and well-structured way, particularly in the introduction, literature review, methodology, results, and discussions in order to achieve the research objectives. Additionally, the discussion section is not exhaustive enough to provide a good attempt to explain many of the study results. 

**Abstract**

 Background – This section should discuss the existing problems of self-diagnosis in the health care system. Why non-invasive screening approach proposed in this study? How this approach is able to overcome the existing self-diagnosis problems? Please include the main objective(s) or research question(s) of this study in this section. The proposed machine learning techniques have to be specifically stated in the methods section. The results section should discuss the result of the combination techniques for each level of classification. 

**Introduction**

 This section should include the following information:

- What are the existing problems in self-diagnosis in the health care system?
- Why is a non-invasive screening approach proposed in this research?
- How is the non-invasive screening approach proposed can overcome these problems?
- What are the existing non-invasive screening approach that has been proposed to solve existing problems in self-diagnosis in the health care system?
- What are the weaknesses of existing methods?
- How is the proposed non-invasive screening approach, able to solve the existing problems in self-diagnosis in the health care system?
- What are the techniques that have been proposed to extract facial features, especially for the health care system? The weaknesses of existing techniques. How is the proposed technique can overcome the current weaknesses? 
- Please include the main objective(s)/research questions of this study in this section.
- Include a brief description of the methodology and classification methods used in this study.

**Literature review/Background study**

 In this section, discussion on the following should be included:

- The major problems in self-diagnosis in healthcare system
- Why non-invasive screening approach suitable to overcome the self-diagnosis problem in the health care system?
- What are the existing methods proposed to overcome these problems? Should discuss the advantages and disadvantages of the existing methods.
- Why are machine learning approaches introduced in solving self-diagnosis problems in the health care system?
- Discuss the advantages of proposing a machine learning approach in solving self-diagnosis problems in the health care system

 

**Methodology**

  

•Discussion about what are the outputs for each method in each classification level. What are the features being extracted? How are these outputs used from one level to another level?
- Discussion about how to obtain the dataset? How are the training and testing datasets divided? 

**Results**

 This section should present the following results:

- without combination methods/ single method
- with combination methods

**Discussions**

 The author(s) should discuss/relate/compare the results with prior relevant studies. Explanation how the performance of the proposed techniques. Provide justification for the performance.

Is the work clearly and accurately presented and does it cite the current literature?

Yes

If applicable, is the statistical analysis and its interpretation appropriate?

Not applicable

Are all the source data underlying the results available to ensure full reproducibility?

Yes

Is the study design appropriate and is the work technically sound?

Yes

Are the conclusions drawn adequately supported by the results?

Partly

Are sufficient details of methods and analysis provided to allow replication by others?

Partly

Reviewer Expertise:

Machine learning, information system, AI

I confirm that I have read this submission and believe that I have an appropriate level of expertise to confirm that it is of an acceptable scientific standard, however I have significant reservations, as outlined above.

# Peer review: Reviewer response for version 1

**DOI:** 10.5256/f1000research.76503.r121344

**Published:** 2022-02-14

**Authors:** Prabina Kumar Meher

**Affiliations:** 1 ICAR-Indian Agricultural Statistics Research Institute, New Delhi, Delhi, India

The authors developed a machine learning-based approach for predicting the health status of an individual by using the image data of their face. Below are my comments for further improvement of the article.

1. The authors must clearly describe how the features were generated from the image dataset.
2. The deep learning method convolutional neural network (CNN) is one of the most appropriate methods for prediction using image data. The authors should employ the CNN for the same and compare the accuracy with that of machine learning algorithms.
3. The author should perform a comparative analysis with the existing methods to claim the superiority of the method.
4. The author must try to establish an online prediction tool for the real use of the developed approach.
5. More work related to this subject must be discussed.

Is the work clearly and accurately presented and does it cite the current literature?

Yes

If applicable, is the statistical analysis and its interpretation appropriate?

Partly

Are all the source data underlying the results available to ensure full reproducibility?

Partly

Is the study design appropriate and is the work technically sound?

Partly

Are the conclusions drawn adequately supported by the results?

Yes

Are sufficient details of methods and analysis provided to allow replication by others?

No

Reviewer Expertise:

Application of Machine learning for prediction/classification using biological data in agricultural and biomedical science.

I confirm that I have read this submission and believe that I have an appropriate level of expertise to confirm that it is of an acceptable scientific standard, however I have significant reservations, as outlined above.

# Response

**Published:** 2022-02-16

**Authors:** Tee Connie

**Affiliations:** Multimedia University, Malaysia

Dear Reviewer,

 Thank you very much for your time and efforts in reviewing our manuscript “Non-Invasive Health Prediction from Visually Observable Features”. According to your valuable comments and suggestions, more analysis has been conducted. In this response letter, we list the specific concerns and questions raised by the reviewer and provide our itemized response.

**Point 1: **The authors must clearly describe how the features were generated from the image dataset.

**Response 1: **We thank the reviewer for the careful review and comment. We understand that it is important to describe how the features were generated from the images. In the study, four feature extraction methods namely local binary pattern (LBP), Principal Component Analysis (PCA), Linear Discriminant Analysis (LDA), and Gabor filter were applied. The details how the features are extracted from the image have been added.

**Point 2: **The deep learning method convolutional neural network (CNN) is one of the most appropriate methods for prediction using image data. The authors should employ the CNN for the same and compare the accuracy with that of machine learning algorithms.

**Response 2: **This is a very good suggestion. In fact, we had also performed experiments using CNN on the image data. However, as the scope of the paper is more towards the analysis and comparison of conventional feature extraction and classification methods rather than the deep learning approach, we did not include the details for CNN in the paper.

 Anyway, to better illustrate the use of CNN on the health dataset, we provide some information about the experiments using CNN in this response letter here.

**Point 3: **The author should perform a comparative analysis with the existing methods to claim the superiority of the method.

**Response 3: **We thank the reviewer for the suggestion. It is important to provide a comparative analysis with the existing methods to claim the superiority of the method. To better illustrate the performance of the proposed methods as compared to state-of-the-art techniques, we have added a new section in Experiments.

**Point 4: **The author must try to establish an online prediction tool for the real use of the developed approach.

**Response 4: **We thank the for the suggestion. It is advantageous to establish an online prediction tool for the real use of the developed approach and we will consider this in our future endeavors.

**Point 5: **More work related to this subject must be discussed.

**Response 5: **Thank you for the suggestion. In addition to the related works that have been discussed in the Literature Review section in the paper, more studies related to the use of face images for health prediction are provided in the article​​​​​​.

# Peer review: Reviewer response for version 1

**DOI:** 10.5256/f1000research.76503.r94202

**Published:** 2021-09-14

**Authors:** Andrews Samraj

**Affiliations:** 1 Department of Computer Science and Engineering, Mahendra Engineering College, Namakkal, Tamil Nadu, India; 2 Mahendra Engineering College, Namakkal, Tamil Nadu, India

Non-invasive health prediction is the essential need of the hour due to the pandemic situation in health care. The approach and modeling using image data set of human faces with sound health and with illness are taken for research and the findings were presented in this work.

 Though the testing accuracies are low, an increase in the number of samples may help in improving.

 The modifications suggested:

1. In methods, the two-level classification has to be depicted clearly.
2. Tables are not visible. Improve font size
3. Results and discussion should only explain the results, assumptions, and parameters.  So explain the variants in the Methods section.

Is the work clearly and accurately presented and does it cite the current literature?

Yes

If applicable, is the statistical analysis and its interpretation appropriate?

Not applicable

Are all the source data underlying the results available to ensure full reproducibility?

Partly

Is the study design appropriate and is the work technically sound?

Yes

Are the conclusions drawn adequately supported by the results?

Yes

Are sufficient details of methods and analysis provided to allow replication by others?

Yes

Reviewer Expertise:

Image processing, Non invasive signal and image analysis, Soft cyborgs, Wearable Technology, Bionics, AI-ML

I confirm that I have read this submission and believe that I have an appropriate level of expertise to confirm that it is of an acceptable scientific standard.
